# RMTSE: A Spatial-Channel Dual Attention Network for Driver Distraction Recognition

**DOI:** 10.3390/s25092821

**Published:** 2025-04-30

**Authors:** Junyi He, Chang Li, Yang Xie, Haotian Luo, Wei Zheng, Yiqun Wang

**Affiliations:** 1 Faculty of Information Science, Huxi Campus, Chongqing University, Chongqing 400044, China; 20230191@stu.cqu.edu.cn (J.H.); zw3475@cqu.edu.cn (W.Z.);; 2 State Key Laboratory of Intelligent Vehicle Safety Technology, Chongqing 400023, China

**Keywords:** driver distraction recognition, vision transformer, channel attention mechanism, transfer learning

## Abstract

Driver distraction has become a critical factor in traffic accidents, necessitating accurate behavior recognition for road safety. However, existing methods still suffer from limitations such as low accuracy in recognizing drivers’ localized actions and difficulties in distinguishing subtle differences between different behaviors. This paper proposes RMTSE, a hybrid attention model, to enhance driver distraction recognition. The framework introduces a Manhattan Self-Attention Squeeze-and-Excitation (MaSA-SE) module that combines spatial self-attention with channel attention mechanisms. This integration enables simultaneous enhancement of discriminative features and suppression of irrelevant characteristics in driving behavior images, improving learning efficiency through focused feature extraction. We also propose to employ a transfer learning strategy utilizing pre-trained weights during the training process, which further accelerates model convergence and enhances feature generalization. The model achieves Top-1 accuracies of 99.82% and 94.95% on SFD3 and 100-Driver datasets, respectively, with minimal parameter increments, outperforming existing state-of-the-art methods.

## 1. Introduction

According to the definition by the US-EU Bilateral ITS Technical Task Force [1], “driver distraction” refers to “the diversion of attention from activities critical for safe driving to a competing activity”. It is widely acknowledged that driver distraction significantly increases the risk of traffic accidents [1]. The advancement of intelligent technologies has introduced additional factors contributing to driver distraction, such as mobile phone usage for calls or text messaging while driving. Data from the World Health Organization’s 2023 Global Status Report on Road Safety [2] reveal an estimated 1.19 million road traffic fatalities in 2021. Therefore, developing real-time recognition of distracted driving behaviors coupled with effective assessment and warning mechanisms holds substantial potential for reducing accident rates caused by driver distraction and enhancing safety for both drivers and passengers.

The comprehensive survey by Tan et al. [3] demonstrates that current primary methods for driver distraction recognition can be categorized into (1) vision-based methods and (2) non-visual data integration and analysis methods. Among these, research utilizing RGB modal data collected by in-vehicle cameras constitutes a crucial component of vision-based approaches, benefiting from advantages including ease of data collection, low implementation costs, and rich semantic/contour information extraction capabilities. Recent advancements in deep learning have empowered improved Convolutional Neural Networks (CNNs), Long Short-Term Memory (LSTM), and Faster Region-based Convolutional Network (Faster R-CNN) models as robust tools for visual recognition tasks, leading to numerous studies on driver distraction recognition, exemplified by FRNet [4], IDC-Bi-LSTM-Att [5], CAT-CapsNet [6], and DD-RCNN [7]. However, existing deep learning-based approaches still face limitations such as inadequate global feature capture and non-parallelizable training processes. The Vision Transformer (ViT), leveraging its unique global modeling mechanism, fully utilizes GPU parallel computing capabilities while effectively capturing global features and inter-feature relationships. This architecture has demonstrated impressive performance on driver distraction recognition datasets, as evidenced by studies including [8,9]. However, traditional ViTs lack the inductive biases inherent in CNNs, thus requiring large amounts of data for learning. This results in ViTs generally underperforming CNNs on smaller datasets. To address this, it is necessary to introduce additional mechanisms that explicitly model ViT’s strong focus on specific regional features critical to task objectives, thereby enhancing the model’s learning efficiency when trained with equivalent data inputs. Meanwhile, compared to lightweight models, ViT requires longer training time to achieve acceptable recognition accuracy, which not only increases the demand for computational resources but also makes deployment more challenging in practical applications. Specifically, the deployment of ViT-based systems in real-world vehicular environments faces critical challenges including but not limited to the following: (1) hardware compatibility constraints for embedded deployment in vehicles with limited computational power, (2) real-time processing requirements for timely distraction alerts, and (3) significant dependency on large-scale diverse training data to maintain robustness across varying demographic characteristics and driving environments. Therefore, how to reduce training time while ensuring the model’s generalization performance and improve the efficiency of utilizing driver distraction behavior data under equivalent parameter counts remains crucial for enhancing the practicality of ViT-based methods in real-world tasks.

The contributions of this paper are summarized as follows:

(1) To achieve superior recognition accuracy on popular large-scale driver distraction datasets, we propose the RMTSE model, which integrates a decomposable self-attention mechanism with explicit spatial priors and a channel attention mechanism (CAM). This design endows the model with robust global spatial information modeling capabilities while enhancing its focus on inter-channel information flow through channel weight recalibration.

(2) We introduce a transfer learning strategy for driver distraction recognition tasks. This approach enables the model to acquire extensive low-level and general feature representations from larger datasets, which are unattainable when relying solely on driver distraction-specific datasets. Subsequent fine-tuning further empowers the model to achieve exceptional characterization of overall driver behavioral features. By leveraging transferable pre-trained weights, the proposed model attains competitive accuracy within a short training period and supports an extension to other driver distraction recognition datasets.

(3) We conduct comprehensive performance comparisons with state-of-the-art models implemented using mainstream methods on identical datasets. The RMTSE model demonstrates outstanding results on both the SFD3 and 100-Driver datasets, achieving higher average accuracy than numerous advanced approaches. These empirical outcomes validate the superiority and feasibility of the proposed method.

## 2. Related Works

### 2.1. Traditional Driver Distraction Recognition Methods

Traditional driver distraction recognition primarily follows two technical approaches: hardware-based sensing and visual analysis. Methods relying on physical sensors directly collect operational data through devices such as steering wheel angle sensors and pedal pressure sensors, offering advantages in high temporal resolution and rapid response. Early computer vision approaches employed combinations of Haar features and AdaBoost cascade classifiers to achieve coarse-grained detection of driver states via in-vehicle cameras.

Li et al. [10] proposed a multimodal framework for monitoring driver distraction by integrating vehicle dynamics, visual behaviors, and auditory cues. The methodology comprises three key components: (1) analyzing operational patterns through vehicle motion parameters extracted from CAN bus signals, focusing on steering wheel dynamics, speed variations, and braking patterns; (2) assessing driver attention via computer vision techniques to track head orientation, eye movement patterns, and gaze characteristics, incorporating personalized calibration to address individual behavioral differences; (3) detecting verbal interactions through audio signal processing. The framework employs scenario-aware preprocessing to filter out specific driving conditions such as low-speed maneuvers and sharp turns, complemented by data imputation techniques to handle sensor anomalies. By innovatively synchronizing multimodal sensor streams and implementing adaptive thresholds for behavior recognition, this approach improves monitoring reliability in complex driving scenarios while establishing a scalable fusion architecture for driver state analysis.

For handcrafted visual feature extraction methods, Seshadri et al. [11] introduced a driver phone usage detection approach using naturalistic driving face videos from the SHRP2 [12] dataset. The authors employed the Supervised Descent Method (SDM) for real-time tracking of 49 facial landmarks, extracting regions of interest (ROIs) near the ears via fixed-area cropping. They combined Histogram of Oriented Gradients (HOG) features with raw pixel features, comparing the performance of AdaBoost, SVM, and random forest classifiers. Experiments validated the method’s effectiveness on the SHRP2 dataset, encompassing both static and dynamic driving scenarios, and established the first benchmark test on this emerging dataset.

However, hardware-based sensing approaches suffer from limitations such as high hardware deployment costs and the inability to recognize limb movements. Meanwhile, early computer vision analysis methods, constrained by the representational capacity of handcrafted features, exhibit elevated error rates in detecting subtle expressions like eye closures and yawns.

### 2.2. Deep Learning-Based Driver Distraction Recognition Methods

Traditional methods exhibit heavy reliance on manual intervention for data annotation and feature engineering, which restricts the generalization capabilities of models. With the rise of deep learning, end-to-end feature learning and automated feature extraction have gradually emerged as dominant trends. As emphasized in [13], significant advancements in deep learning (DL) research in recent years have created substantial technical evolution opportunities for subsequent breakthroughs in DL-based driving behavior monitoring methods [13,14]. The adoption of deep learning methodologies not only overcomes traditional dependencies on handcrafted features but also enhances system robustness, enabling more precise and efficient detection of distracted behaviors in complex driving scenarios.

For spatial feature extraction, Convolutional Neural Networks (CNNs) represent a critical technology for driver state detection. By leveraging end-to-end learning of localized motion patterns such as eye closure, mouth movements, and head pose variations, deep CNNs (e.g., VGG [15], ResNet [16]) or multi-stage processing frameworks can effectively capture subtle features of distracted behaviors (e.g., phone usage, eating). Lightweight CNN architectures, such as MobileNet [17] and EfficientNet [18], further optimize computational efficiency, rendering them suitable for real-time monitoring on embedded in-vehicle systems [13]. For instance, Duan et al. [4] proposed FRNet, which introduces a Feature Reorganization Block to redistribute spatial features into depth dimensions. This innovation significantly compresses feature map volumes while reducing memory access and multiply-accumulate (MAC) operations. Additionally, the authors designed an ultra-lightweight atypical backbone network combined with pixel-level analysis to optimize feature reshaping strategies, achieving an effective balance between model efficiency and accuracy. However, despite CNNs’ local inductive bias enabling effective modeling of spatial locality, their capacity to capture long-range dependencies and global features remains inherently limited.

To address this limitation, Recurrent Neural Networks (RNNs) and their variants (e.g., Long Short-Term Memory (LSTM) networks and Gated Recurrent Units (GRUs)) have been introduced into driver behavior analysis to focus on modeling long-range dependencies in sequences of distracted driver images. Wang et al. [5] proposed a driver distraction recognition model (IDC-Bi-LSTM-Att) integrating Improved Dilated Convolutional Neural Networks (ID-CNN), Bidirectional LSTM (Bi-LSTM), and an attention mechanism. This model employs stepped dilation convolutions to extract multi-scale spatial features, expanding the receptive field to 19 × 19 to enhance the capture of subtle motions. The bidirectional LSTM is utilized to model long-range dependencies in driver behavior image features, while a lightweight attention module dynamically allocates feature weights to suppress background interference. The model achieves high accuracy on the StateFarm and custom-built datasets, respectively. However, LSTMs exhibit higher computational complexity than basic RNNs and insufficient support for parallelization, limiting their scalability for long-sequence data processing.

In the realm of deep learning-based driving behavior monitoring, Faster R-CNN [19] and its variants have demonstrated remarkable potential. Lu et al.’s DD-RCNN framework [7] achieves precise driver action recognition through multi-level architectural innovations. Built upon Faster R-CNN, this method introduces deformable and dilated residual blocks (DDRbs) to address feature extraction challenges for small, irregularly shaped targets (e.g., phones, cigarettes). Deformable convolution dynamically adjusts sampling positions to capture target deformation features, while dilated convolution expands receptive fields to enhance fine-grained feature representation. A channel-spatial dual-dimensional attention mechanism is embedded in high-level backbone features to amplify critical region responses through feature reweighting. To mitigate redundant region proposals, a region proposal optimization network (RPON) employs binary classification for efficient high-quality candidate screening, significantly improving computational efficiency without compromising accuracy. Experimental results demonstrate that this architecture, combined with alternating optimization training and hard sample mining strategies, achieves substantial performance gains over baseline models in multi-class behavior recognition, validating its effectiveness and robustness in complex driving scenarios.

The Transformer [20] was initially proposed for machine translation tasks. Unlike traditional RNNs and LSTMs, the Transformer model does not rely on sequential processing of input sequences but instead processes the entire input in parallel, significantly enhancing training efficiency. Simultaneously, the self-attention mechanism enables each input element to reference all other positions in the sequence during output generation, endowing the model with superior representational capacity. To adapt the Transformer’s strengths to computer vision (CV) tasks, researchers have developed a series of Vision Transformers (ViTs) [21,22,23,24]. Leveraging its self-attention mechanism for powerful global modeling, ViT demonstrates unique advantages in driving monitoring tasks. By dividing driver distraction images into patches and encoding them as sequential features, the Transformer can model global dependencies across regions (e.g., correlations between hand movements and steering wheel operations). For instance, Vision Transformer (ViT) and its variants have been applied to analyze driver posture, in-vehicle object interactions, and road environment information, substantially improving detection robustness in complex scenarios [25,26]. However, compared to traditional CNN-based visual models, ViT exhibits limitations in model architecture and feature representation. The original ViT model is sensitive to input image sizes and struggles to fully exploit global image information, significantly impacting classification performance. To address this, researchers have proposed hybrid architectures that integrate CNN-derived structures such as multi-scale pyramids and residual connections into ViT. These hybrid models leverage CNNs to extract low-level local features (e.g., gestures, facial landmarks) and subsequently fuse global contextual information via self-attention, thereby enhancing adaptability to input image dimensions and feature extraction capabilities [25]. Additionally, the absence of CNN-like inductive biases necessitates substantially larger datasets for effective ViT training, amplifying data dependency requirements. To address the lack of inductive biases in ViT, we designed a MaSA-SE module that integrates Manhattan self-attention [24] with SE-Net [27] employing the channel attention mechanism and introduced a transfer learning strategy for model training.

## 3. Methodology

**Figure 1:**
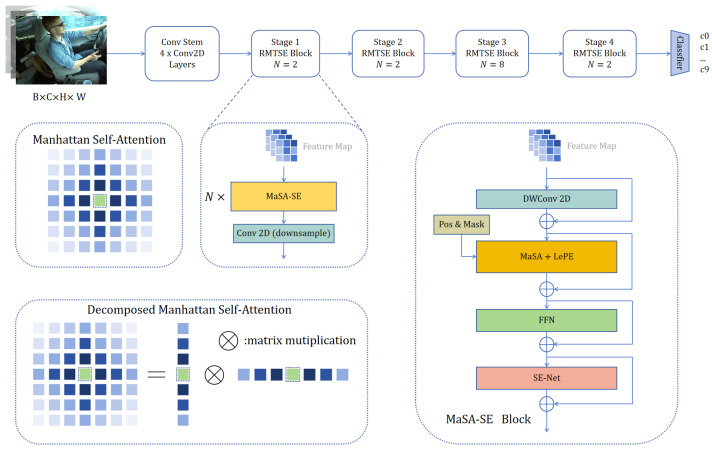
The feature construction process of RMTSE. The raw image is first fed into a convolutional backbone to generate preliminary feature maps, then sequentially processed through four consecutive stages. MaSA, within different stages, adopts decomposed or non-decomposed (original) forms depending on specific requirements.

The proposed RMTSE architecture aims to achieve precise driver distraction recognition. The effectiveness of integrating channel attention mechanisms into ViT stems from their ability to model nonlinear relationships between feature channels, complementing the self-attention mechanism. While ViT achieves global spatial dependency modeling through self-attention mechanisms, its core computational process fundamentally performs indiscriminate aggregation of features across all spatial positions, which lacks explicit evaluation of feature significance along the channel dimension. The output features from each ViT block often exhibit information redundancy across channels, where certain channels may contain task-irrelevant noise or secondary characteristics. SE-Net addresses this through adaptive channel weight learning, performing channel-wise recalibration of intermediate features to optimize information structure for subsequent layers. Particularly in deep networks, as inter-channel disparities in high-level semantic features intensify, SE-Net’s channel attention mechanism enhances category-relevant feature responses, enabling more efficient computational resource allocation during feature processing. A detailed schematic of the RMTSE model is shown in the accompanying Figure 1. The convolutional backbone initially extracts low-level feature information such as edges and basic shapes from input images. The MaSA-SE module prioritizes interactions between global spatial features and channel-wise information. Within this module, decomposable self-attention with Manhattan distance-based decay cooperates with a learnable channel attention mechanism, first modeling spatial dependencies and subsequently reassigning feature map importance across channels. When specific channels contribute minimally to feature representation, their information may be suppressed by the channel attention mechanism.

### 3.1. Overall Architecture

Figure 1 illustrates the overall architecture of RMTSE. RGB images are first processed through a convolutional backbone containing four 3 × 3 convolutional layers to capture preliminary features and perform downsampling. The resultant feature maps are then fed into four consecutive stages for hierarchical feature construction from low-level to high-level representations. Each stage contains *N* MaSA-SE modules followed by a 3 × 3 convolutional layer with stride 2 for further downsampling. Within each MaSA-SE module, decomposable self-attention with customized decay computation is implemented. A channel attention module is positioned at the module’s end to holistically recalibrate the output. To reduce computational complexity, decomposed MaSA is applied in the first two stages, while the original MaSA operates in the latter two stages to enhance complex feature extraction. Finally, the output features are passed to a classifier for prediction.

### 3.2. MaSA-SE Module

Traditional Transformer-style attention mechanisms lack CNN-like inductive biases for local features, rendering the model incapable of distinguishing differences between image patches. To address this, positional encoding (PE) must be explicitly introduced. Notably, in driver behavior images, the contributions of different spatial regions to distraction recognition vary significantly. Due to camera perspective constraints, critical regions for distinguishing driver features are often concentrated in limited areas. Even within these regions, distinct features exhibit varying degrees of influence on specific behavior recognition. To ensure the model captures essential features, we aim to focus attention on regions more conducive to final classification. For this purpose, we propose integrating a channel attention mechanism to further amplify or suppress information at specific locations based on PE, thereby enabling the model to enhance the learning capacity of key features.

#### 3.2.1. Manhattan Distance-Decayed Self-Attention

Manhattan Self-Attention (MaSA), initially proposed in RMT [24], introduces an attention mask that decays according to the Manhattan distance between pixel pairs. Compared to conventional self-attention mechanisms that indiscriminately process embedded 2D patches, MaSA incorporates richer spatial priors while maintaining linear computational complexity for global information modeling. The original MaSA can be formulated as Equation (1):

(1) $$\mathrm{MaSA}\left(X\right)=\left(\mathrm{Softmax}\left(QK^{T}\right)⊙D^{2d}\right)V$$

where $D^{2d}$ denotes the decay matrix computed based on Manhattan distances between token pairs in the 2D plane, defined as:

(2) $$D_{nm}^{2d}=\gamma^{\left|x_{n}-x_{m}\right|+\left|y_{n}-y_{m}\right|}$$

The decomposed MaSA is expressed as Equation (3). By recalculating decay matrices along horizontal ($D_{nm}^{H}=\gamma^{\left|y_{n}-y_{m}\right|}$) and vertical ($D_{nm}^{W}=\gamma^{\left|x_{n}-x_{m}\right|}$) directions

(3) $$\begin{array}{cc} \; & Attn_{H}=\mathrm{Softmax}\left(Q_{H}K_{H}^{T}\right)⊙D^{H},\\ \; & Attn_{W}=\mathrm{Softmax}\left(Q_{W}K_{W}^{T}\right)⊙D^{W},\\ \; & \mathrm{MaSA}\left(X\right)=Attn_{H}{\left(Attn_{W}V\right)}^{T} \end{array}$$

Each self-attention module in MaSA-SE employs Locally-Enhanced Positional Encoding (LePE) [23]. Unlike the absolute positional encoding in the original ViT [21], LePE utilizes convolutional operations to capture relative positional information, which is directly applied to the output of the self-attention module to enhance spatial awareness. The feature maps processed by MaSA are subsequently fed into Feed-Forward Networks (FFN) for further refinement.

#### 3.2.2. Learnable Channel Weight Recalibration Module

At the end of each MaSA-SE module, we embed an SE-Net [27] module to capture channel attention from the input feature maps. This module compresses spatial dimension information via global average pooling to generate channel descriptors, followed by a two-layer fully connected (FC) network with nonlinear activation to model inter-channel dependencies. Finally, adaptive weights dynamically recalibrate feature channels. Specifically, the module comprises “Squeeze” and “Excitation” phases. For an input feature map $X\in R^{H\times W\times C}$, the Squeeze operation is formulated as Equation (4):

(4) $$z_{c}=\frac{1}{H\times W}\sum_{i=1}^{H}\sum_{j=1}^{W}u_{c}\left(i,j\right)$$

The Excitation operation follows:

(5) $$s=\sigma \left(W_{2}\delta \left(W_{1}z\right)\right)$$

(6) $${\tilde{x}}_{c}=s_{c}\cdot u_{c}$$

where $W_{1}\in R^{C\times C/r}$, $W_{2}\in R^{C/r\times C}$, r=16 (reduction ratio), δ(·) denotes ReLU, and σ(·) denotes sigmoid. Although this module introduces minimal parameters, it delivers substantial performance improvements, as demonstrated in the experimental section.

#### 3.2.3. Depthwise Separable Convolution and Regularization

As illustrated in the figure, each MaSA-SE module begins with a Depthwise Separable Convolution (DwConv) layer to preliminarily process input feature maps for patch embedding. The inductive bias inherent in convolutional layers enables the model to better retain local spatial information. Unlike standard convolutions, DwConv decomposes the convolution process into Depthwise Convolution and Pointwise Convolution, which reduces computational complexity and parameter count while enhancing model efficiency and inference speed. Furthermore, Layer Normalization (applied within the FFN module) and DropPath are integrated into the MaSA-SE module. At the end of each stage, Batch Normalization (implemented in the downsampling module) is adopted for model regularization. These mechanisms collectively improve generalization capability and ensure training stability.

### 3.3. Transfer Learning Strategy

Transfer learning assumes that different tasks share inherent correlations, enabling knowledge acquired from one task to be transferred to another related task. Through pre-training, models can learn rich feature representations from large-scale datasets. In driver distraction behavior recognition tasks, fine-tuning allows the model to adjust its weights according to the target task’s data distribution, effectively leveraging knowledge from pre-training to accelerate convergence and enhance performance. ImageNet-1k [28], the most widely used subset of the ImageNet dataset, contains 1000 categories covering common objects in daily life (e.g., animals, vehicles, tools, natural scenes). These objects exhibit abundant contour and structural information, enabling models pre-trained on this dataset to develop robust capabilities in constructing and expressing low-level visual features and general patterns—attributes critical for characterizing holistic driver distraction behaviors.

Consequently, we adopt ImageNet-1k as the pre-training dataset. Specifically, we initialize the RMTSE model (excluding the SE-Net modules and classification head) with weights pre-trained on ImageNet-1k using the RMT-T [24] architecture. Since the final output for driver distraction recognition datasets involves 10 classes, we redesign and initialize the classification head. The SE-Net modules, characterized by minimal parameters and a focus on inter-channel relationships within input feature maps, are trained alongside the model during fine-tuning. For the SFD3 dataset, to enhance efficiency and consider that the initial three stages of the backbone primarily extract low-level features, we freeze all parameters in the convolutional backbone and the first three stages (except SE-Net modules). Only the SE-Net modules, the final stage (employing original MaSA), and the classification head are trained from scratch. For the 100-Driver dataset, all parameters are unfrozen to facilitate fair comparisons with other methods. The cross-entropy loss function is adopted for optimization.

## 4. Experiments

### 4.1. Dataset Description

The State Farm Distracted Driver Detection (SFD3) dataset [29], provided for a Kaggle competition, aims to identify driver distraction behaviors through computer vision techniques. It contains 22,424 annotated images categorized into 10 classes: c0: Safe driving, c1: Texting with right hand, c2: Calling with the right hand, c3: Texting with left hand, c4: Calling with the left hand, c5: Operating the radio, c6: Drinking, c7: Reaching behind, c8: Adjusting hair or makeup, c9: Talking to passengers. All images were captured by a single-view camera mounted in a vehicle from a single perspective, with a resolution of 640 × 480 pixels in RGB color format.

The 100-Driver dataset [30] is designed for the Distracted Driver Classification (DDC) task. It comprises over 470,000 driving behavior images from 100 drivers captured by four cameras, with a resolution of 1920 × 1080 pixels and 22 distinct driver behavior classes. This dataset features cross-modal, cross-vehicle, and cross-view characteristics. Compared to SFD3, the 100-driver dataset exhibits greater diversity in lighting conditions and camera perspectives, introducing additional challenges for accurate distraction recognition.

**Figure 2:**
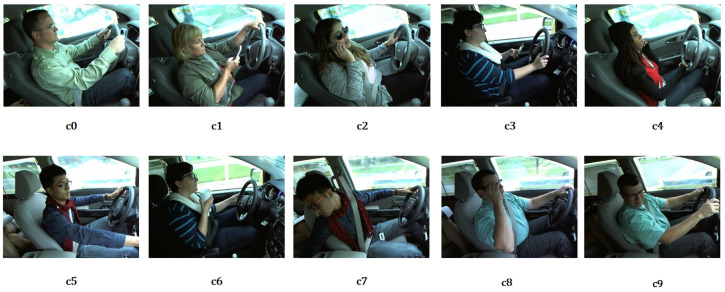
Example images of ten classes from SFD3.

**Figure 3:**
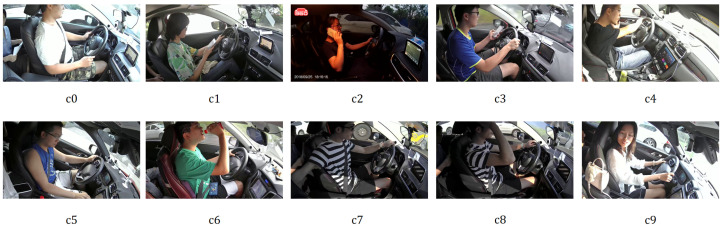
Example images of ten classes from 100-Driver.

Example images from the SFD3 and 100-driver datasets are shown in Figure 2 and Figure 3, respectively. A detailed comparison of class distributions is provided in Table 1.

### 4.2. Experiment Setting

During implementation, the deep learning framework PyTorch 2.5.1 was employed to construct the model. All experiments were conducted on Linux operating systems. For the SFD3 experiments, the system utilized an Intel Core i9-10980XE CPU @ 3.00 GHz and an NVIDIA GeForce RTX 3090 GPU with 24 GB of VRAM, both of which source from Chongqing, China. Experiments on the 100-Driver dataset were performed on a system equipped with an Intel Xeon Platinum 8481C CPU and an NVIDIA GeForce RTX 4090 D GPU with 24 GB of VRAM, both of which source from Chongqing, China. Input images were uniformly resized to 224 × 224 pixels, with a batch size of 64 and an initial learning rate of 1 $\times \;10^{-6}$. The AdamW optimizer was adopted for training. All results were obtained after training for 50 epochs, with data augmentation techniques applied. Specific hyperparameter configurations are detailed in Table 2.

### 4.3. Experiment Results on SFD3

The SFD3 dataset was randomly partitioned into 80% for training and 20% for testing. The proposed model achieved a top-1 accuracy of 99.82% on the SFD3 dataset. Comparative analyses were conducted against various models employing CNN, LSTM, and ViT methodologies. Detailed performance metrics are presented in Table 3.

**Figure 4:**
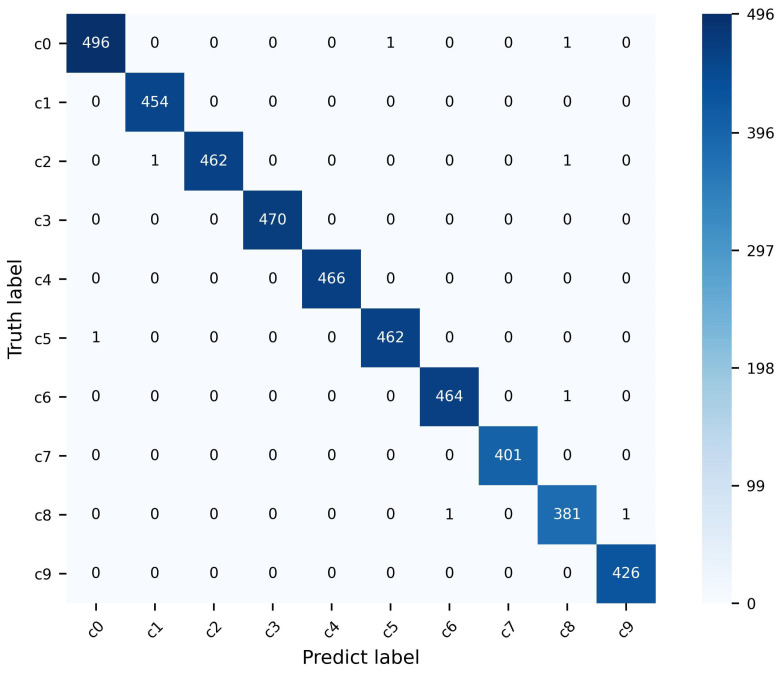
Confusion matrix of RMTSE on the SFD3 dataset. Only sporadic misclassifications are observed across a few classes.

Figure 4 illustrates the confusion matrix of the proposed model on the SFD3 validation set. Notably, the model exhibits robust discrimination capabilities across all classes, demonstrating its strong potential for precise identification of driver distraction behaviors.

### 4.4. Experiment Results on 100-Driver

The SFD3 dataset can be approximately regarded as a subset of the 100-Driver dataset in terms of driver behavior categories. Following the methodology of [33], we extracted 10 categories from the original 22 classes in 100-Driver to align with those in SFD3. Additionally, we utilized all daytime images from camera 4 (cam4), whose placement closely resembles the setup in SFD3. This approach introduces similarities in data categorization and feature distribution, facilitating comparative analysis with experiments on SFD3. The training and test sets were partitioned using the conventional 8:2 split ratio as specified in the 100-Driver study [30]. The proposed model achieved a 94.95% top-1 accuracy on the 100-Driver dataset. Comparative evaluations against multiple models demonstrate that RMTSE achieves significant advantages in accuracy, parameter count, and computational complexity over conventional ViT and traditional CNN-based methods. Compared to RMT-T, our proposed model achieves effective enhancement in recognition accuracy while introducing very few additional parameters. Detailed metrics are provided in Table 4.

**Figure 5:**
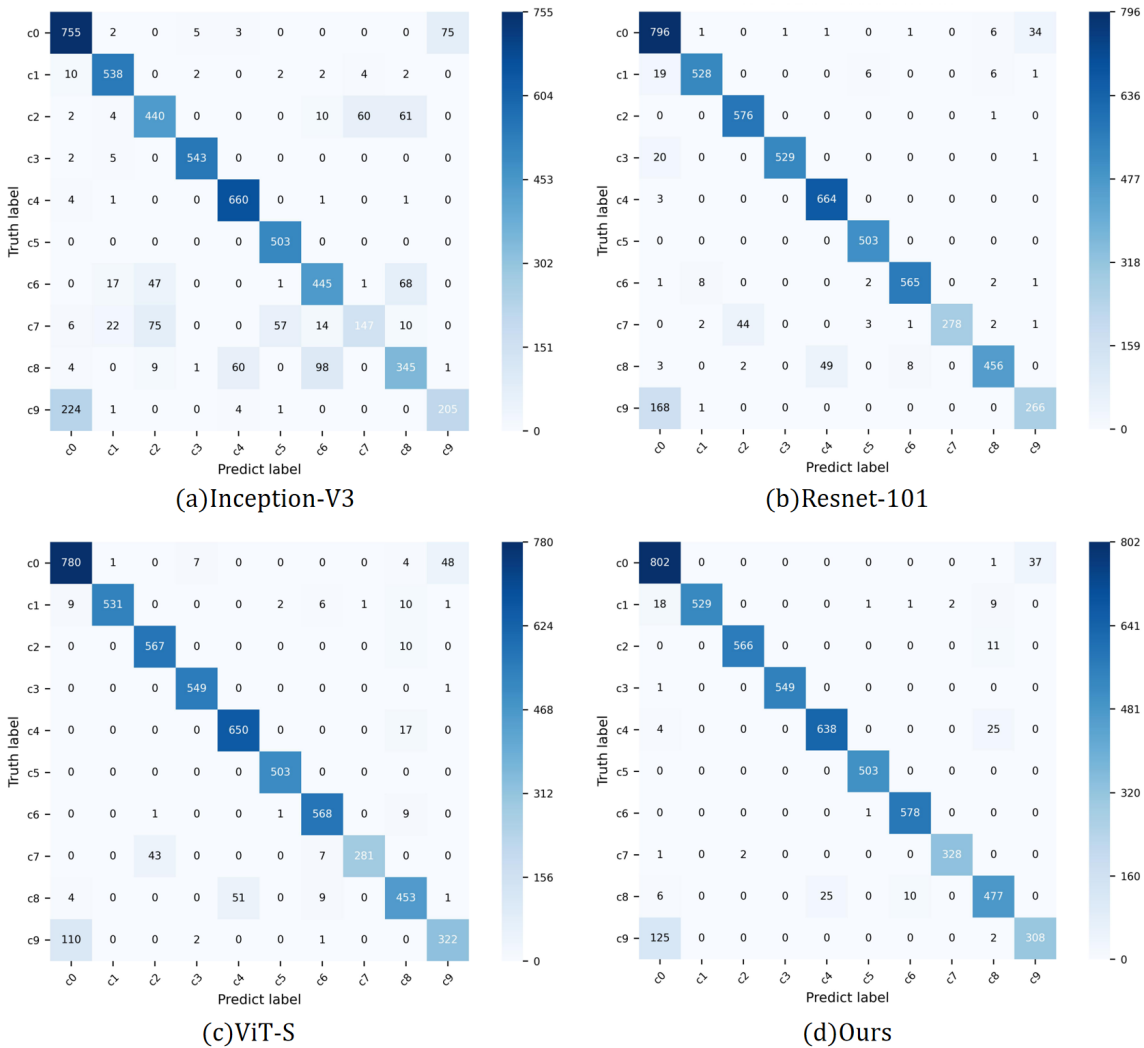
Confusion matrices of the selected models on the 100-Driver dataset. RMTSE demonstrates the highest recognition accuracy, while ResNet-101 and ViT-S exhibit higher confusion between c7 and c2, as well as c8 and c4. Inception-V3 shows the most severe misclassification. All models display significant confusion between c9 and c0.

In Figure 5, we present the confusion matrices of multiple models on the 100-Driver validation set. Analysis reveals that the proposed RMTSE model exhibits superior overall recognition capability across all categories compared to the other benchmarked models.

### 4.5. Ablation Study

#### 4.5.1. Ablation Study on SE-Net

**Figure 6:**
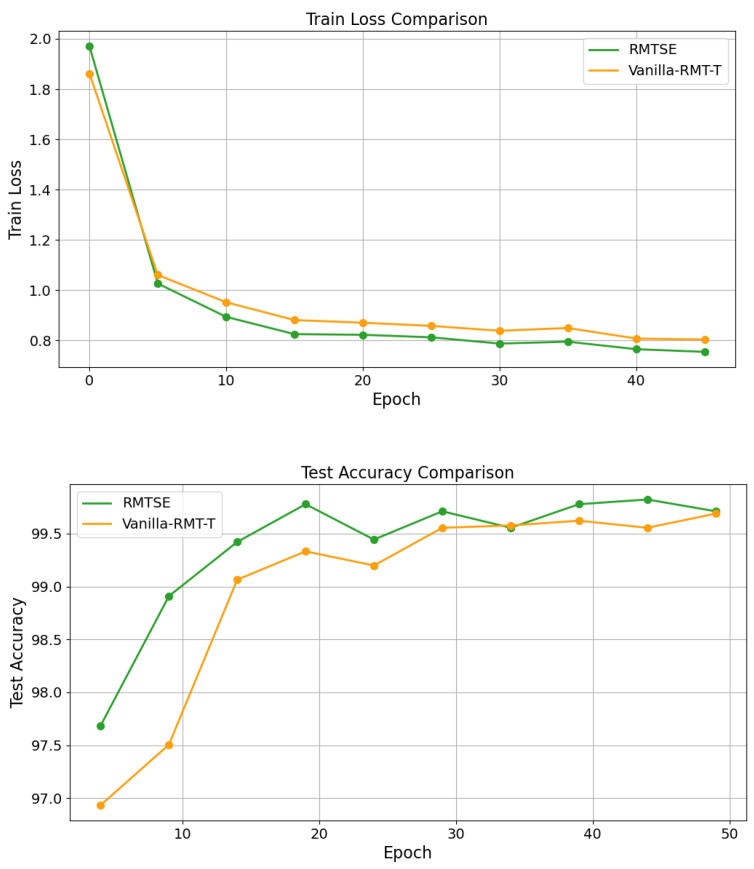
Comparison of training loss (**top**) and top-1 test accuracy (**bottom**) with and without the SE-Net module.

As shown in Figure 6, compared to the original RMT-T without the SE-Net module, RMTSE exhibits faster convergence in training loss and achieves lower loss plateaus. The right part illustrates the Top-1 test accuracy trajectories of RMTSE and RMT-T across training epochs. It is evident that after several initial epochs, RMTSE consistently achieves higher top-1 test accuracy at equivalent epochs compared to RMT-T. These results validate that the SE-Net module effectively recalibrates features through its channel attention mechanism.

#### 4.5.2. Ablation Study on Transfer Learning

**Figure 7:**
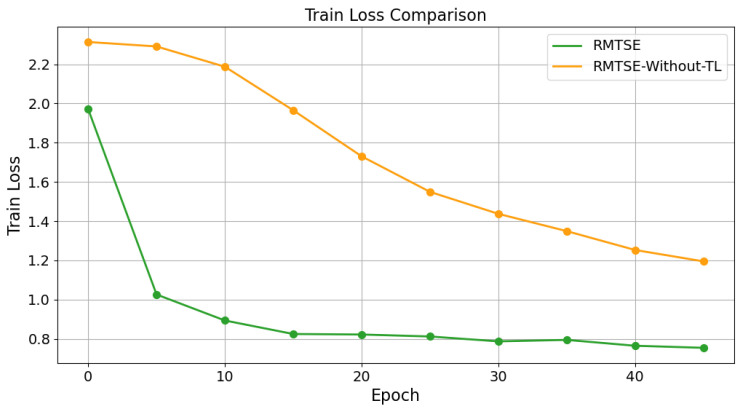

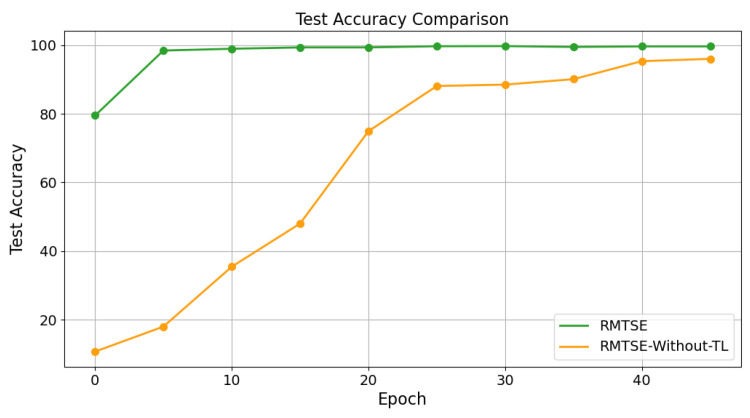
Comparison of training loss (**top**) and top-1 test accuracy (**bottom**) with and without transfer learning (TL).

Figure 7 presents a global comparison of training dynamics between the RMTSE model employing transfer learning (TL) and the model trained from scratch without pre-trained weights, evaluated through training loss and Top-1 test accuracy. Experimental results demonstrate that the TL-enabled model achieves convergence to 97.68% Top-1 test accuracy within 5 epochs. In contrast, the model without TL first exceeds 97% (reaching 97.13%) only at the 46th epoch. This indicates that the TL-enabled model bypasses the need to relearn elementary features and generic patterns. Instead, it leverages pre-trained knowledge of fundamental feature representations to directly assemble global characteristics of driver distraction behaviors. Compared to training from scratch, the TL approach significantly reduces training time while enhancing recognition accuracy for driver distraction behaviors.

### 4.6. Model Visualization

**Figure 8:**
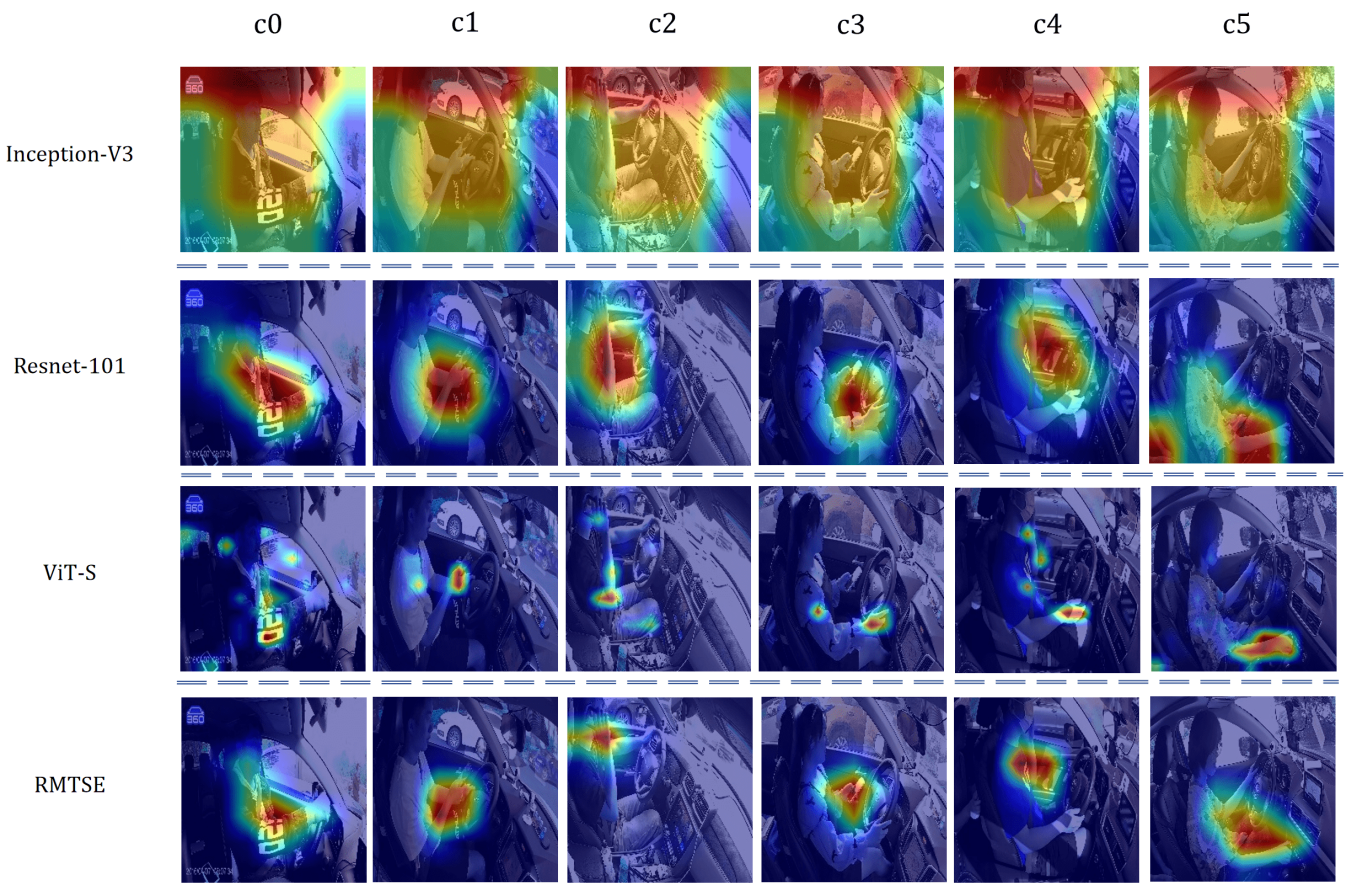
GradCAM visualization results of selected models across different scenarios, where each row corresponds to a different model. RMTSE consistently exhibits continuous and precise attention to critical features in all scenarios.

To visualize the model’s attention and verify whether it focuses on semantically relevant image features, we employ Gradient-weighted Class Activation Mapping (Grad-CAM). Grad-CAM generates heatmaps to highlight regions in input images that most strongly influence predictions for specific classes. Figure 8 illustrates the attention patterns of four distinct models on example images from categories c0 to c5 in the 100-Drivers dataset. The six example images cover hand movements in different locations. For instance, in the c3 category (Texting with the left hand), ResNet-101’s attention shifts toward the central region rather than the critical left-hand area, while ViT-S disperses attention and erroneously captures features from the right hand. In contrast, RMTSE precisely localizes the left hand and mobile phone regions, aligning closely with the core characteristics of the behavior, while maintaining spatial separation from irrelevant regions. Across other examples, RMTSE consistently focuses on key limb regions with high inter-class discriminative power. Moreover, its activation maps exhibit spatial continuity, enabling effective capture of local contextual information around critical features, thereby enhancing differentiation from other classes.

## 5. Limitation

**Figure 9:**
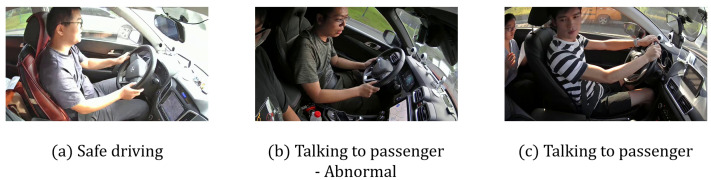
Comparison of driver behaviors. From left to right: (**a**) c0: Safe driving; (**b**) Abnormal c9: Talking to passenger missing key features; (**c**) Standard c9 image. The absence of critical features and inter-image similarity is a primary cause of severe confusion.

Although the RMTSE model demonstrates superior recognition performance on both datasets, analysis on the confusion matrix (Figure 5) reveals that all tested models exhibit pronounced confusion between c0 (Safe driving) and c9 (Talking to passengers) in 100-Driver dataset, a phenomenon not observed in the SFD3 dataset. Upon inspecting the training data, we identified that this discrepancy primarily stems from instances in the 100-Driver dataset where drivers in c9 lacked distinctive head-turning movements—a critical feature for distinguishing this class from others. Without this feature, the only discriminative characteristic between c9 and c0 becomes subtle differences such as slight mouth opening in certain frames. Such nuanced variations are inherently challenging for models to capture, resulting in substantial misclassification. A detailed comparative visualization is shown in Figure 9. Future work may address this limitation by integrating dedicated mechanisms for micro-expression detection to enhance discriminative capability.

Furthermore, while the spatial-channel dual attention mechanism and hierarchical feature extraction architecture proposed in this study effectively enhance recognition accuracy, they remain constrained by the inherent mechanisms and characteristics of the ViT model, resulting in demanding computational resource requirements. As shown in the Table 4, the RMTSE model still exhibits a computational complexity of 2.651 GFLOPs, which may pose compatibility challenges in real-time demanding scenarios such as in-vehicle embedded deployment. Although parameter efficiency optimization has been achieved through the SE-Net module, it remains necessary to strike a balance between accuracy and computational overhead in practical applications with limited edge computing resources. To address this limitation, the knowledge distillation techniques could be investigated in future research to transfer knowledge from larger-scale models to compact variants. This methodology aims to preserve comparable recognition performance while substantially reducing model complexity, thereby enhancing deployment adaptability for resource-constrained in-vehicle embedded computing systems.

## 6. Conclusions

The proposed RMTSE model achieves high-precision recognition of driver distraction behaviors through its spatial-channel dual attention mechanism and hierarchical feature extraction architecture. The incorporation of transfer learning further endows the method with rapid deployment capability and scalability, improves the recognition accuracy of driver distraction behavior, and reduces dependency on the data volume of driver distraction datasets. Extensive experiments on the SFD3 and 100-Driver datasets validate the model’s efficacy, achieving Top-1 accuracies of 99.82% and 94.95%, respectively, surpassing numerous state-of-the-art methods. The ablation study reveals that the SE-Net module enhances parameter efficiency by recalibrating features through channel-wise attention.

## Figures and Tables

**Table 1:** Driver behavior classes and datasets.

Class Description	SFD3	100-Driver
c0: Safe driving	2489	4455
c1: Texting with right hand	2267	3284
c2: Calling with right hand	2317	3400
c3: Texting with left hand	2346	3140
c4: Calling with left hand	2326	3479
c5: Operating the radio	2312	2512
c6: Drinking	2325	2882
c7: Reaching behind	2002	2068
c8: Adjusting hair or makeup	1911	2402
c9: Talking to passenger	2129	2485

**Table 2:** Hyperparameter configuration.

Parameters	Values
Input size	224 × 224
Epochs	50
Batch size	64
Optimizer	AdamW
Initial learning rate	1 $\times \;10^{-6}$
MixUp (α)	0.8
DropPath rate	0.1

**Table 3:** Performance evaluation of various models on SFD3.

Models	Top-1 Acc. (%)
DenseNet121 [31]	94.83
InceptionV3 [32]	92.90
IDC-Bi-LSTM-Att [5]	95.84
DDR-ViT-finetuned [9]	97.50
RMT-T [24]	99.71
RMTSE (Ours)	99.82

**Table 4:** Performance evaluation of various models on 100-Driver.

Models	Top-1 Acc. (%)	Params (M)	FLOPs (G)
InceptionV3 [34]	82.86	21.8	2.845
ResNet101 [16]	92.86	42.52	7.83
ViT-S [21]	93.60	21.67	4.25
RMT-T [24]	94.26	13.32	2.65
RMTSE (Ours)	94.95	13.46	2.651

## Data Availability

The raw data supporting the conclusions of this article will be made available by the authors upon request. All datasets used are publicly available.
